# Developing National Cancer Registration in Developing Countries – Case Study of the Nigerian National System of Cancer Registries

**DOI:** 10.3389/fpubh.2015.00186

**Published:** 2015-07-30

**Authors:** Elima E. Jedy-Agba, Emmanuel A. Oga, Michael Odutola, Yusuf M. Abdullahi, Abiodun Popoola, Peter Achara, Enoch Afolayan, Adekunbiola Aina Fehintola Banjo, Ima-Obong Ekanem, Olagoke Erinomo, Emmanuel Ezeome, Festus Igbinoba, Christopher Obiorah, Olufemi Ogunbiyi, Abidemi Omonisi, Clement Osime, Cornelius Ukah, Patience Osinubi, Ramatu Hassan, William Blattner, Patrick Dakum, Clement A. Adebamowo

**Affiliations:** ^1^Department of Non-Communicable Disease Epidemiology, London School of Hygiene and Tropical Medicine, London, UK; ^2^Institute of Human Virology Nigeria, Abuja, Nigeria; ^3^The Marlene and Stewart Greenebaum Cancer Centre, Institute of Human Virology, University of Maryland School of Medicine, Baltimore, MD, USA; ^4^Federal Teaching Hospital Gombe, Gombe, Nigeria; ^5^Lagos State University Teaching Hospital, Ikeja, Nigeria; ^6^Federal Medical Centre Keffi, Keffi, Nigeria; ^7^University of Ilorin Teaching Hospital, Ilorin, Nigeria; ^8^Lagos University Teaching Hospital, Lagos, Nigeria; ^9^University of Calabar Teaching Hospital, Calabar, Nigeria; ^10^Federal Teaching Hospital, Ido Ekiti, Nigeria; ^11^University of Nigeria Teaching Hospital, Enugu, Nigeria; ^12^National Hospital Abuja, Abuja, Nigeria; ^13^University of Port Harcourt Teaching Hospital, Port Harcourt, Nigeria; ^14^University College Hospital Ibadan, Ibadan, Nigeria; ^15^Ekiti State University Teaching Hospital, Ado-Ekiti, Nigeria; ^16^University of Benin Teaching Hospital, Benin City, Nigeria; ^17^Nnamdi Azikiwe University Teaching Hospital, Nnewi, Nigeria; ^18^Federal Ministry of Health, Abuja, Nigeria

**Keywords:** cancer registration, Nigeria, population-based cancer registries, cancer incidence, cancer

## Abstract

The epidemiological transition in sub-Saharan Africa (SSA) has given rise to a concomitant increase in the incidence of non-communicable diseases including cancers. Worldwide, cancer registries have been shown to be critical for the determination of cancer burden, conduct of research, and in the planning and implementation of cancer control measures. Cancer registration though vital is often neglected in SSA owing to competing demands for resources for healthcare. We report the implementation of a system for representative nation-wide cancer registration in Nigeria – the Nigerian National System of Cancer Registries (NSCR). The NSCR coordinates the activities of cancer registries in Nigeria, strengthens existing registries, establishes new registries, complies and analyses data, and makes these freely available to researchers and policy makers. We highlight the key challenges encountered in implementing this strategy and how they were overcome. This report serves as a guide for other low- and middle-income countries (LMIC) wishing to expand cancer registration coverage in their countries and highlights the training, mentoring, scientific and logistic support, and advocacy that are crucial to sustaining cancer registration programs in LMIC.

## Introduction

Sub-Saharan African (SSA) countries are now undergoing an epidemiological transition with increasing incidence of non-communicable diseases (NCD) occurring alongside persistence of communicable diseases epidemic (1, 2). These two epidemics are also feeding off each other with infections like HIV, Hepatitis B and C, *Helicobacter pylori* leading to increased incidence of certain cancers and rising prevalence of other risk factors for cancer (3–5). In addition, increased life expectancy, reduced mortality from infectious diseases, increasing urbanization, and westernization leading to changes in lifestyle, dietary, obstetrics, and physical activity practices in this region have been associated with increased risk of NCD including cancer (3, 6, 7). The HIV/AIDS epidemic and prevalent oncogenic infections are also contributing significantly to SSA’s rising cancer burden (8, 9).

With this has come the realization that information from cancer registries is vital for monitoring the incidence, prevalence and mortality of cancer, the effectiveness of national cancer prevention and cancer control initiatives, resource allocation, and public policy related to cancer control (10). Although recent literature report an increase in cancer incidence in developing countries, poor vital and cancer registration remains a widespread problem such that reliable information on cancer incidence and mortality is scarce (5, 11–13). Cancer registries are veritable tools for collecting accurate and complete information on cancer incidence, prevalence, and mortality in a given geographical location and they can be used to conduct research, plan and implement cancer control, allocate resources for treatment and prevention, and other public health program planning (14). Cancer registries serve as a starting point for basic research and etiological studies and therefore play a crucial role in cancer prevention. Where cancer registries collect information on follow-up, they can be used to assess the survival of cancer patients and evaluate the efforts made at improving survival.

Despite these acknowledged utilities of cancer registration, cancer registries are not yet an integral part of cancer control in most low- and middle-income countries (LMIC). In those LMIC where resources exist and cancer control policies have been formulated, there is often a lack of political will to rigorously implement these policies (15). The implication is that policy makers in LMIC lack information about the population burden of cancer and its health, economic, and political implications (10, 13).

In this paper, we report the history of cancer registration in Nigeria and, the conceptualization as well as recent implementation of a system for representative nation-wide cancer registration in Nigeria. We highlight the key challenges encountered in implementing this strategy and how they were overcome. This report may provide guidance for other LMIC as they implement cancer registration programs.

## History of Cancer Registration in the World and Africa

The registration of cancer cases began with several unsuccessful attempts at cancer surveys in the United Kingdom in 1728, Germany in 1900, and Netherlands and Spain in 1902 and 1908 (16). After several sporadic attempts at population-based cancer registration in Germany in 1926, USA, Denmark, England, and Canada in 1940s (17), the need for the establishment of cancer registries throughout the world was recommended to the World Health Organization (WHO) by leading experts in the field of cancer control (17). A few years later, the WHO established a subcommittee mandated to proffer recommendations for the establishment of cancer registries.

The specialized arm of the WHO that deals with cancer, the International Agency for Research on Cancer (IARC) was formed in 1965 and the following year, the International Association of Cancer Registries (IACR) was founded (16). The IACR and IARC through their activities have promoted the development of cancer registration in many developing regions including SSA. Cancer registration in Africa began in 1950s with registries in South Africa, Uganda, Nigeria, and Zimbabwe (Table 1).

**Table 1:** **Earliest cancer registries in sub-Saharan Africa (45)**.

Registry/country	Year registry was founded	Founder
Johannesburg, South Africa (SA)	1953	Higginson and Oettle
Kampala, Uganda	1954	Davis, Templeton
Cape Town, South Africa	1956	Muir Grieve
Lorenco Marques, Mozambique	1956	Prates
Ibadan, Nigeria	1960	Edington
Bulawayo, Zimbabwe	1963	Skinner
Durban, South Africa	1964	Schonland and Bradshaw

These African registries contributed cancer incidence data to the WHO/IARC Cancer in five Continents (CIV) publications; [Mozambique: Lorenco Marques; Nigeria: Ibadan; South Africa: Johannesburg, Bantu and Uganda: Kyadondu (Volume 1), South Africa: Cape Province; South Africa: Johannesburg; Nigeria: Ibadan; Zimbabwe Bulawayo (Volume 2), Nigeria: Ibadan; and Zimbabwe: Bulawayo (Volume 3)] until the late 1970s and 1980s when an economic recession and accompanying “brain drain” spread throughout Africa. Since then other registries that have contributed to subsequent CIV volumes include registries Dakar Senegal, Mali, The Gambia, and Harare Zimbabwe.

Although many African registries submit data for the CIV publication, data quality issues usually result in a good number of submissions being screened out. However, over the last decade, there has been a gradual reawakening of the need for cancer registries that can generate high-quality data with a resultant increase in the number of IARC acknowledged PBCRs (member registries of the African Cancer Registry Network) in Africa (Table 2) (18).

**Table 2:** **Population-based cancer registries in sub-Saharan Africa in 2013 (Source: www.afcrn.org) (18)**.

Country (Number of PBCRs)	Registries	Country Population (m = million) (t = thousand)	Population covered by registry	Year of conception
Botswana^a^ (1)	Botswana National Cancer Registry	2,024,787 m	2,024,787 m	1999
Brazzaville^a^ (1)	Registre des Cancers de Brazzaville	4,337,051 m	4,337,051 m	1995
Cote d’Ivoire (1)	Registre des Cancers d’Abidjan	19,839,750 m	3,772,230 m	1994
Ethiopia (1)	Addis Ababa Cancer Registry	91,728,849 m	3,384,569 m	2011
The Gambia^a^ (1)	Gambia National Cancer Registry	1,791,225 m	1,791,225 m	1986
Ghana (1)	Kumasi Cancer Registry	25,366,462 m	2,035,064 m	2012
Guinea (1)	Registre de Cancer de Guinea	11,451,273 m	1,656,300 m	1990
Kenya (2)	Eldoret Cancer Registry	43,178,141 m	894,179 t	1999
	Nairobi Cancer Registry		3,138,369 m	2001
Malawi (1)	Malawi Cancer Registry	15,906,483 m	1,895,973 m	1993
Mauritius^a^ (1)	Mauritius Cancer Registry	1,291,456 m	1,291,456 m	1993
Mozambique (1)	Registre de Cancro de Beira	25,203,395 m	457,799 t	2005
Namibia^a^ (1)	Namibia Cancer Registry	2,259,393 m	2,259,393 m	1995
Niger (1)	Registre des Cancers du Niger	17,157,042 m	1,011,227 m	1992
Nigeria (3)	Abuja Cancer Registry	168,833,776 m	1,406,239 m	2009
	Calabar Cancer Registry		647,458 t	2004
	Ibadan Cancer Registry		2,549,265 m	1960
Rwanda (1)	Rwanda Cancer Registry	11,457,801 m	–	1991
Seychelles^a^ (1)	Seychelles National Cancer Registry	88,303 t	88,303 t	2008
South Africa (3)	South African Children’s Cancer Group (SACCSG) Tumor	52,274,945 m	15,800,000 m	1987
	Registry South African National Cancer Registry (Pathology-based)		52,274,945 m	1986
	PROMEC Cancer Registry, Eastern Cape		1,300,000 m	
Tanzania (1)	Tanzania Cancer Registry	47,783,107 m	4,364,541 m	2009
Uganda (1)	Kampala Cancer Registry	36,345,860 m	2,010,000 m	1951
Zimbabwe (1)	Zimbabwe National Cancer Registry	13,724,317 m	1,468,766 m	1985

*^a^Registries with national coverage*.

## Cancer Registration in Nigeria

Nigeria is the most populous country in Africa. With a population of approximately 168 million people, it represents over 50% of the population of the West African sub-region and slightly <20% of the population of Africa (19). The country was recently reclassified by The World Bank as a lower middle income country with a total GDP of $568.5 billion, GDP per capita of $2,688 and a total health expenditure of 5.3% (19). Life expectancy in the country also increased from 47 years in 2001 to 52 years in 2011 (19). Improvement in cancer registration in Nigeria is therefore likely to contribute significantly to the proportion of Africans covered by this method of cancer surveillance.

In Nigeria, cancer registration began in 1960 with the first cancer registry located within the Pathology Department of the University College Hospital Ibadan, in South Western Nigeria. Cancer incidence data from this registry were included in the first three volumes of Cancer Incidence in five continents (CIV) for the time periods 1960–1962, 1960–1965, and 1960–1969 (20). Edington and Mclean published the first paper on the cancer profile in Nigeria with data from the Ibadan cancer registry in the British Journal of Cancer in 1965 (21). All through this period, the Ibadan Cancer Registry was the only population-based cancer registry in Nigeria contributing data to CIV.

However, between the seventies and end of 2000s, the Ibadan Cancer Registry stopped submitting data to CIV. The reasons for this include severe economic, political, and social upheavals that affected Nigeria during this period. The severe economic retrogression led to high levels of emigration by educated citizens including doctors, many of whom had trained abroad and returned to the countries where they trained or to the Middle East (22). This brain drain, along with marked reduction in funding to public health care systems, led to the demise of many cancer registries, stymied the development of new ones and retarded efforts at integrating the cancer registries into a national cancer data reporting system.

During the four decades interregnum when there was no reliable information on cancer incidence, prevalence, and mortality in the country, most published cancer data were based on case series, medical and pathology departments’ records, and mortality reports from autopsies (23). These are not valid sources of cancer incidence data because they do not provide information on cancer incidence in the general population (24). Most of these data sources were limited in scope and coverage often leading to overrepresentation of easy to biopsy tumors and may be more reflective of the nature of clinical practice, personnel, and infrastructure in the reporting institutions (25). There was also declining practice of autopsy in Nigeria throughout this period (26). Over-interpretation and misunderstanding of the data from these sources has led to significant misunderstanding of the cancer incidence in Nigeria and similar LMIC to date.

The first attempt to coordinate the activities, provide training and mentoring, as well as ensure a uniform standard of reporting for all cancer registries in Nigeria led to the establishment of the National Headquarters of Cancer Registries in Nigeria (NHCRN) by the Nigerian Federal Ministry of Health (FMOH) in 1990s. The NHCRN led by an Executive Chairman, Professor of Surgery Toriola Feyisetan Solanke at the University College Hospital (UCH) and the College of Medicine, University of Ibadan, Ibadan, Nigeria, was initially located at the Department of Surgery and later in the Department of Radiotherapy, UCH, Ibadan, Nigeria.

The NHCRN implemented training programs, conducted public advocacy and supplied resource materials to several cancer registries in the country. Data from different cancer registries were collated and some of this was published in 1998 (27). For several years, the data published by NHCRN were the reference information on cancer incidence data in Nigeria. However, the death of the pioneer Executive Chairman of the NHCRN, continuing economic difficulties and failures in the public health systems in Nigeria conspired to make the NHCRN largely non-functional from 2002. During this period and afterwards, IARC continued to support the Ibadan, Ile-Ife and Calabar registries and other cancer registration activities in Nigeria even as the quality of the data produced often fell short of the standard required for publication in CIV.

## Implementing a National System of Cancer Registration in Nigeria

Although cancer registration began decades ago in Nigeria, progress over the past 50 years has been slow, patchy, and halting. Previous efforts at achieving quality population-based cancer registries have not been sustained. This poor progression to highly functional population-based cancer registration has been mainly as a result of lack of financial and institutional support for cancer registries, ignorance of the need for cancer registration and poorly trained staff and registry personnel. With the advent of democratic rule in Nigeria, improvements in public health financing and management, there has been a renewal of interest in cancer registration.

In 2009, the Nigerian FMOH, Society of Oncology and Cancer Research of Nigeria (SOCRON – http://socron.net/) and the Institute of Human Virology Nigeria (IHVN) conceptualized the Nigerian National System of Cancer Registries (NSCR) as a method for generating cancer incidence data that covers different sections of the country with support from the Fogarty International Center and the National Cancer Institute of the United States’ National Institutes of Health and the Marlene and Stewart Greenebaum Cancer Center, University of Maryland School of Medicine. The main objective of the NSCR is to provide training, capacity development, mentoring, technical, and scientific support to cancer registries in Nigeria to enable them attain population-based cancer registration status and generate high-quality cancer incidence, treatment, and survival data for the country.

The NSCR coordinates the activities of the cancer registries and generates aggregate national cancer incidence, treatment, and survival data; disseminate the data to relevant government agencies for use in policy formulation and resource allocation; to scientists conducting cancer research; and to the public for education, awareness, and advocacy purposes. The NSCR also advocates for cancer registration in the country, increase awareness of cancer, advocate and publish locally relevant cancer data. In order to achieve these objectives, NSCR works to strengthen existing cancer registries, establish new registries through the provision of training, mentoring, computer hardware and software, and provide support for data management and analysis. The NSCR has brought more visibility to the growth and expansion of cancer registries in Nigeria. Through the establishment of the NSCR, there is a more cohesive and synergized approach to cancer registration in the country. In the past, sporadic attempts by many individual cancer registries had been made that did not yield tangible gains. With collation of data from various registries across different geo-political zones, we are able to show variations in cancer incidence, which are helpful in tailoring cancer prevention and control programs.

Through this collaboration, Nigerian cancer registry data are now more easily accessible to researchers with the consent of the individual cancer registries. The NSCR also helps prevent data loss at cancer registry sites. In instances where data loss is encountered; the data have easily been retrieved from the NSCR database. Through the NSCR, linkages are made with other databases to enable Nigerian registry data reach a wider and more relevant audience.

## Activities of the Nigerian National System of Cancer Registries

### Training

**Figure 1:**
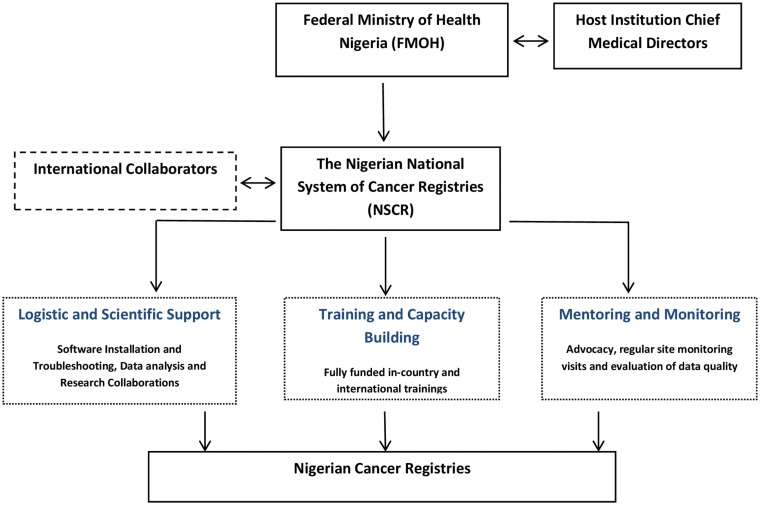
**Framework for effective cancer registration in developing countries**.

In its first few years, NSCR focused on providing training and building the capacity of existing cancer registries with the aim of improving the quality of data generated by these registries. These trainings were provided in collaboration with several international partners including the IARC and International Prevention Research Institute (iPRI) and funded by the United States National Institutes of Health, Greenebaum Cancer Center of the University of Maryland School of Medicine, Baltimore and the Nigerian FMOH (Figure 1).

National System of Cancer Registries has trained and re-trained over 70 registry directors, cancer registrars, and data clerks to date. Training programs have focused on principles of cancer registration, basic cancer epidemiology, the use of CanReg – the cancer registration software developed by IARC (Initially version 4 and later version 5), coding and classification schemes, data quality control, cancer staging and grading, data presentation, management and analysis, and preparation of reports.

### Mentoring and monitoring

An important factor in successful capacity building is mentoring that empowers trainees to internalize and practice newly acquired skills under supervision. Therefore, NSCR places a high level of importance on continuing mentoring and supervision of cancer registries in Nigeria through telephone, e-mail, and face-to-face mentoring visits. From 2009 to 2013, the NSCR conducted 51 mentoring and monitoring visits to 17 cancer registries in different parts of the country with most of these registries visited more than once. Registries are chosen for site visits based on specific criteria outlined below:

1. New registries: newly established registries were visited to ensure sufficient capacity to perform cancer registration exists. During the visit, advocacy visits were paid to institutional authorities to canvass support for cancer registration at the site, local infrastructure including office space, staffing, expertise, budgetary provisions, and local cooperation necessary for cancer registration is evaluated.
2. HBCRs with potential to become PBCRs over a short period of time: HBCRs that have demonstrated sufficient ability to commence population-based cancer registration are visited. Registries that have had a satisfactory level of case ascertainment per year, high-quality data, well trained registry staff; institutional support, formidable organizational structure, and willingness to upgrade are considered to be potential PBCRs. During these visits, a definite coverage population is mapped out, potential data sources are reached, logistics for transportation and data management are arranged; training on coding/classification and the use of CanReg software is provided with the sole aim of commencing sustainable population-based cancer registration.
3. Existing PBCRs. Registries that have attained PBCR status are visited periodically for evaluation of registration methods and data quality. Completeness of coverage is evaluated by visiting established data sources and looking for possible new sources of cancer information within the coverage area. Utilization of medical records index cards is encouraged to ensure complete case ascertainment. Accuracy of registration is also evaluated using the CanReg software and the NSCR team members conduct data validation by re-abstracting and recoding a random sample of cases for comparison with registry data.

These monitoring and mentoring visits provide an opportunity to evaluate data quality and ensure best practices in cancer registration according to international standards. Reports from these visits are forwarded to the Cancer Control Unit of the FMOH and have brought the attention of the government to gaps in the cancer registries system.

### Advocacy

Successful implementation of cancer registration requires cooperation of many stakeholders in the health and non-health care sectors whose cooperation must be assiduously cultivated. These include private and public oncology care and pathology services providers, medical records, vital statistics, community leaders, patients, and government officials. To obtain effective coordination of cancer registration, all stakeholders involved must work together.

Advocacy at the level of the hospital is particularly instrumental in ensuring sustainability of the program. Engaging heads of institutions, heads of medical records and cancer registry directors is crucial to generating much needed local support for the cancer registries. Hospitals should be mandated to set up Cancer Registry Management Committees that can oversee the function and administration of cancer registries. These committees can advocate to hospital management, government, and society on behalf of cancer registries. Often identified as integral to the success of any cancer registry is the leadership of the registry. The director of a cancer registry is expected to play a supervisory role and be involved in monitoring the activities of the cancer registrars particularly as regards data abstraction and recording. The scientific/medical qualification of registry directors while important is secondary to their ability to engage all stakeholders with skill, diplomacy, and tact.

Registries in institutions where the leadership is interested in cancer registration have often fared better than those where this is lacking. In most institutions, the cancer registry staff is deployed from the medical records department and may be rotated out of the registry after they have undergone several cancer registry trainings with new persons brought in with no knowledge of cancer registration principles or use of the CanReg software. This scenario can be prevented by securing the support of the head of the medical records department.

The NSCR provides information through quarterly newsletters in print and online to all institutions within the country. These newsletters serve as an important way of disseminating information about cancer registration activities, provide information about upcoming trainings and encourage registries that are making progress by profiling such registries. They also encourage weaker registries to improve and thus create a healthy form of in-country competition among registries.

### Peer-reviewed publications

The Nigerian cancer registry databases were critically assessed and analyzed in collaboration with researchers at the iPRI Lyon France. Following the review of the registry databases and exclusion of unsuitable submissions, findings from the Abuja and Ibadan population-based cancer registries as well as 11 HBCRs where published in 2 separate journal articles in 2012 (23, 28). The PBCRs reported on the age-standardized incidence rates (ASR) of the most common cancers in Nigeria; cancers of the breast (54.3/100,000) and cervix in women (34.5/100,000) and cancers of the prostate in men (19.1/100,000) (23). Information on the number of cases by site and sex, most valid basis of diagnosis as reported by 11 hospital-based cancer registries in Nigeria has also been published (28).

The value of the cancer registration data generated by NSCR is also being extended through collaborations and additional analyses. A highly accessed pilot AIDS-Cancer Registry Match study at selected institutions in Nigeria showed that the SIR (95% CI) for the AIDS Defining Cancers was 5.7 (4.1, 7.2) and 2.0 (0.4, 3.5), for Kaposi Sarcoma (KS) and Cervical Cancer, respectively (29). Comparing these with findings from the United States Age Cancer Match Study by Engels et al. (30); they reported SIR over two time periods 1990–1995 (pre-HAART) and 1996–2002 (Post-HAART). For KS, SIR was 22,100 (pre-HAART) and 3640 (post-HAART) and for cervical cancer SIR 4.2 (pre-HAART) and 5.7 (post-HAART). Engels and colleagues showed dramatic declines in risk of KS but no change in risk of cervical cancer. Compared to the SIR for KS and cervical cancer in Nigeria reported by Akarolo-Anthony et al. (29), the SIR for KS is significantly higher in the United States than in Nigeria, but this does not hold true for cervical cancer. The effect of age heaping on cancer rate estimation and the comparability, diagnostic validity, and completeness in Nigerian cancer registries have also been studied in detail (31).

The NSCR began coordinating activities of cancer registries in Nigeria in 2009. Clearly, more still needs to be done to improve completeness of registry coverage and data quality yet the data produced to date serve as a valid representation of the cancer problem in Nigeria. Efforts have been intensified to ensure continued improvement of the quality and comprehensiveness of data collected in future through regular scheduled two monthly site visits by a member of the NSCR team.

#### Cancer Incidence in 5 Continents Vol. X and GLOBOCAN 2012

The most recent edition of the IARC publication Cancer Incidence in five continents (CIV) was released in 2013 with contributions from eight PBCRs in Africa, four of which were SSA cancer registries of South Africa, Malawi, Zimbabwe, and Uganda. The Ibadan cancer registry submitted data for this publication but its data were not accepted for inclusion owing to failure to meet the criteria for eligibility based on data quality. Other PBCRs in Nigeria did not have data for the time period under consideration for CIV (2003–2007). However, the Abuja, Calabar, and Ibadan population-based cancer registries made submissions that were included in the GLOBOCAN 2012 database of the IARC launched on the 12th of December 2013. Combined estimates from the Ibadan, Abuja, and Calabar registries submission to GLOBOCAN 2012 on the most common cancers and their ASR are presented in Table 3 below. These findings are similar to previous data published in the literature (23).

**Table 3:** **Most common cancers and age standardized incidence rates in Nigeria in 2012 (46)**.

Male	Total cases* (ASR per 100,000)	Female	Total cases* (ASR per 100,000)
Prostate (c.61)	11,944 (30.7)	Breast (c.50)	27,304 (51.1)
Liver (c.22)	7875 (15.2)	Cervix (c.53)	14,089 (29.2)
Non-Hodgkin’s lymphoma (C82–85, c.96)	2328 (3.7)*	Liver (c.22)	4172 (8.2)
Colorectal (c.18–21)	2164 (4.5)	Colorectal (c.18–c.21)	2008 (4.0)
Kaposi sarcoma (c.46)	982 (1.5)	Non-Hodgkin’s lymphoma (c.82-85, c.96)	1778 (2.8)
All sites but skin (c.44)	37,540 (79.5)	All sites but skin (c.44)	64,622 (122.8)

*Combined estimates derived from the Ibadan, Abuja, and Calabar PBCRs.

The PBCRs in Ibadan, Calabar, and Abuja, Nigeria generate data that is comparable to other international PBCR in terms of coding methods used, definitions of incidence dates and multiple primaries. More recently, 3 other population-based cancer registries have been created in Enugu, Sokoto and Ekiti bringing the total number of PBCRs in Nigeria to 6 as at June 2015. An improvement in completeness and data quality is now the primary focus of the NSCR’s mentoring and monitoring effort in order to generate higher quality data that will meet the criteria for inclusion in future volumes of CIV.

The NSCR’s 5-year strategic work-plan (2013–2018) includes activities designed to improve the quality of PBCR in Nigeria; elevate some existing HBCR to PBCR status and create new PBCR to ensure adequate coverage of a geographical, cultural, and economically diverse country like Nigeria; extend the range of data collected to include cancer outcomes; make Nigerian cancer registry data freely available and accessible online for use by researchers and policy makers behind a registration firewall and publication of regular series on Nigerian Cancer Statistics.

## Challenges of Cancer Registration in Nigeria

While the challenges of cancer registration in Nigeria are similar to those in other African countries that have been described in previous literature (32, 33), the large population, limited health care infrastructure, evolution of cancer registration in Nigeria, Nigerian laws and constitutional arrangements, which puts legislation and responsibility for health care on the concurrent legislative agenda of the federal and state government, among other issues create some unique challenges (Table 4). It is inconceivable and unnecessary for Nigeria to have cancer registries in every part of the country, and hence, the conceptualization of a system that envisages a limited number of population-based cancer registries in different regions of the country so that the potential variation across the rather large and populous country can be adequately captured.

**Table 4:** **Major challenges of cancer registration in Nigeria**.

S/No	Challenges identified	Key findings
1.	Management and mentoring of multiple cancer registries	Growing number of cancer registries within network requires more time, technical and financial capability to manage
		Individually tailored interventions to deal with specific challenges at each registry are often required
2.	Funding and institutional commitment	Lack of registry-specific funding
		Logistic and resource constraints
		Rotation of cancer registry staff
		Cancer registration not prioritized by host institutions
		Various competing needs by infectious diseases and other areas for hospital funds
3.	Inadequate education and training	Cancer registrars with little or no computer literacy
		Poor knowledge of cancer registration principles, practices, and CANREG software
		Medical doctors often sent for trainings meant for hospital registry staff
4.	Data collection	Incompleteness of abstraction owing to lack of cooperation from data sources
		Under-reporting
5.	Data quality	Quality indicators, such as DCO%, MV% are quite low
		No mortality data for calculation of mortality incidence ratios (MI ratios)
		Place of residence often inaccurate
		Poor age estimation
6.	Sustainability	Insufficient funding opportunities for cancer registries

### Management and mentoring of multiple cancer registries

Managing the growing number of cancer registries within the NSCR network is not trivial. Cancer registries across the country need to be continuously mentored, monitored, and evaluated with feedback provided on their registration activities. The monitoring and evaluation of this program have often involved local and international experts who provide unbiased assessments of current status and areas in need of improvements. Tailored interventions are then developed for individual cancer registries as recommended following these visits.

The NSCR bridges the gap between the government, local institutions, stakeholders, and the individual registries. However, cancer registration in Nigeria can only be sustained through strong integration of NSCR within the FMOH, engagement at the highest level of leadership in the health sector, evident contribution of cancer registries data to the FMOH for policy making and resource allocation, and continuous advocacy. The development of joint implementation strategies with measurable registry output are also very important components.

### Funding and institutional commitment

While there is widespread recognition of the need for cancer registration and the data they generate, the response at individual institutional levels has been weak. Most institutions are unwilling or unable to support a transition from hospital to population-based cancer registration. There are several reasons for this including increasing use of fee for service models to run health care in Nigeria. Institutions therefore often lack funds to implement public health interventions like cancer registration amidst competition for limited funds by various departments in the hospital, inadequate staff and lack of evident “purchaser” of cancer registration services.

The most common difficulty encountered by cancer registries in Nigeria is inadequate resources needed to carry out their activities. Many cancer registrars and advocates have frequently supplemented cancer registration activities from personal resources but this is not sustainable and often results in sporadic efforts with long intervals between abstractions. Other models for funding cancer registration including use of competitive grants, cooperative agreements, or fee for service models need to be explored by governments and development partners.

### Education and training

Trained personnel are a crucial resource in cancer registration but they are in short supply. Opportunities for training and re-training have also been limited. With the limited support for cancer registries in institutions, support for local and international training of staff is often not forthcoming from the hospitals where the registries are located. The NSCR recognizing this gap, embarked on intensive training of registry personnel over the past few years, nationally and internationally supported by funding from the United States National Institutes of Health’s Fogarty International Center and the National Cancer Institute. However, continuous training and re-training, mentoring, monitoring, and trouble shooting are needed for registry staff as well as registry directors to ensure that best registry practices are upheld.

### Data collection

Lack of cooperation from data sources within a registry’s catchment area adversely affects completeness of the data that the registry can collect. Our experience revealed a plethora of problems encountered with many potential data sources. There is general suspicion of cancer registrars, which may be related to a general low level of trust in the society, lack of a well-established culture of data collection and data management, and general distrust of government and government related activities. There are also concerns about patient confidentiality and how these would be protected given that cancer is still a stigmatizing disease and most patients do not share information about their disease even with close relatives. Some data sources ask to be paid before release of data while some refuse to cooperate for unknown reasons. Government regulations making cancer a *registrable disease* (thus empowering registrars’ access to institutional records) in contrast to being a *notifiable disease* (where the onus is on institutions to report the disease to government authorities as required by law) would increase the authority of cancer registrars and reduce the challenges encountered at health institutions. In many instances, health facilities do not have organized, stored, and accessible medical records of their own. Enforcement of existing regulations on health records, investment in medical records and education of all stakeholders about their value is urgently needed.

### Data quality

Poor quality is a common problem with data from many developing countries. If data quality is low, comparability with other regions is poor and estimates of incidence, mortality and survival are inaccurate. Information on date of birth and age are particularly important to collect in a cancer registry for accurate estimation of age-specific incidence (ASI) and ASR. Age data in Nigeria are not always reliable because many individuals do not know their age and rigorous effort is not made at medical registration to help the individual provide the best estimate through the use of prompts although such methods also have significant limitations. Medical records and health care workers therefore use broad categories, such as “adult” in lieu of the age of patient. With increasing education of the populace and use of vital statistics in many other aspects of life, this problem should diminish over time.

Contact address information of patients is also usually incomplete. It is particularly important to ensure that the address provided by the patient is the “usual” residential address and not a place of temporary abode that is used to access hospital care for duration of treatment. It is not uncommon for patients in Nigeria to temporarily re-locate to addresses in cities where the tertiary institution that can provide cancer treatment is located and give this information when their address is sought. Patients may also be unwilling to give specific and detailed address out of a lack of understanding of the need for such information and lack of trust about how the information may be used. Establishing a system for verifying addresses should be priority for all cancer registries in Nigeria.

In Nigeria, like many other LMIC, there is poor recording of vital statistics. Mortality data are unreliable because not all deaths are reported, a significant proportion of deaths occur at home and the causes are unknown. The deceased are buried in their homes or communities without report to municipal authorities. Death certification or autopsies are not always done so specific causes of death are not reported (34). This lack of ancillary but necessary vital information for cancer registries affects data quality and limits data analysis (18). Evaluating cancer registry data quality using the indices of completeness, validity, comparability, and timeliness has been held back by these gaps in the vital registration system (35–39).

### Sustainability

For cancer registration efforts to be sustained in Nigeria, local ownership is essential. While financial and technical advice provided by international partners is critical, long-term success, and sustainability is a shared responsibility between local stakeholders and the government. Stakeholders must accept the responsibility to establish, sustain, and routinely monitor the system. Clear lines of budgetary support and reporting channels must be established. In order to establish this in Nigeria, a National Cancer Registration Advisory Committee including representatives of the highest level of government, members of the National Cancer Control Committee, representatives of different stakeholders in-country in the country is being established while the NSCR continue to provide relevant guidance, technical and scientific support.

It is essential that after receiving training and support, cancer registries produce data of reasonably good quality that can be used to generate information for cancer control and public policy. In addition, such data can contribute to research on cancer and for application for funding from relevant funding agencies.

### Registry research, ethics, and confidentiality

Cancer registries are notably useful sources of information on the burden of disease in a given population by providing information on incidence, mortality, and survival and less commonly on prevalence and disability adjusted life years (DALYs) (40). Other important uses of cancer registry data include descriptive studies and analytical research into the etiology and risk factors of specific cancers. Although as a widely acclaimed rule, most cancer registries function under strict conditions of confidentiality of medical information, there are conflicting views on use of registry data in research without previous patient consent (40). In registries of some western countries, informed consent is now a prerequisite for recording patient information into a registry database (41). This legislation adversely affects the completeness and validity of a registry’s database and several authorities in the field have stressed that individual patient consent is not feasible and should not be a requirement in any cancer registry (42). In Nigeria, the National Health Research Ethics Committee has determined that expedited approval is sufficient and has provided this for all cancer registration work at all sites in the country thereby ameliorating a need to seek individual approval at each institution. This approach may help in other countries too.

## Conclusion

Central coordination of cancer registration in countries like Nigeria with large population and multiple cancer registries is crucial. This enable collation, aggregation, analysis, and reporting of data, identification of regional and local trends that can spur further epidemiological research and generation of information that can guide government policy and resource allocation. Such evident benefits of cancer registration will spur local support and buy-in.

It is well-established globally that population-based cancer registration is central to cancer registration, epidemiology, and control programs (14, 40, 43). However, we and others have argued that until such registries are established, there is role, albeit limited, for hospital-based cancer registries (28, 44). Their data can be informative and contribute to cancer control policies and resource allocation. Governments need to consider making cancers *registrable* thus empowering cancer registrars to collect relevant information without hindrance from public and private health institutions. Cancer registration must be entrenched in government cancer control policies and initiative and the government must provide guidelines on cancer registrations to all stakeholders in the health sector. Options for funding cancer registration other than through competition for funds allocated to support therapeutic services must be given serious consideration.

Nigeria has recently taken major strides in improving cancer registration. With the implementation of a central coordinating body, the NSCR and its role in implementing and sustaining these efforts, considering that Nigeria’s population is 17–20% that of Africa, high-quality cancer registration from Nigeria will significantly reduce the proportion of Africa that is not currently covered by cancer registration. At least three population-based cancer registries have been established or strengthened and the quality of their data continues to improve. Several hospital-based cancer registries are being supported and mentored to achieve population-based registry status over the next few months. Cancer epidemiology data are being regularly produced, analyzed, and submitted to government for advocacy, planning, policy, and budgetary purposes. Nevertheless, more work needs to be done to expand the scope of data collected, their quality, and their utilization.

## Author Contributions

All these authors contributed equally to the writing of this paper. CA and WB obtained funding for the project, provided critical revisions to the manuscript, and guided all aspects of this paper. All authors read and approved the final manuscript.

## Conflict of Interest Statement

The authors declare that the research was conducted in the absence of any commercial or financial relationships that could be construed as a potential conflict of interest.
